# Recommendations from a consensus development workshop on the diagnosis of fetal alcohol spectrum disorders in Australia

**DOI:** 10.1186/1471-2431-13-156

**Published:** 2013-10-02

**Authors:** Rochelle E Watkins, Elizabeth J Elliott, Amanda Wilkins, Raewyn C Mutch, James P Fitzpatrick, Janet M Payne, Colleen M O’Leary, Heather M Jones, Jane Latimer, Lorian Hayes, Jane Halliday, Heather D’Antoine, Sue Miers, Elizabeth Russell, Lucinda Burns, Anne McKenzie, Elizabeth Peadon, Maureen Carter, Carol Bower

**Affiliations:** 1 Telethon Institute for Child Health Research, Centre for Child Health Research, The University of Western Australia, P.O. Box 855, West Perth, WA 6872, Australia; 2 Discipline of Paediatrics and Child Health, Sydney Medical School, University of Sydney, Sydney, Australia; 3 The Children’s Hospital at Westmead, Sydney, Australia; 4 The George Institute for Global Health, Sydney, Australia; 5 Child and Adolescent Health Service, Department of Health Western Australia, Perth, Australia; 6 Centre for Population Health Research, Curtin University, Perth, Australia; 7 Centre for Chronic Disease, School of Medicine, University of Queensland, Brisbane, Australia; 8 Public Health Genetics, Genetic Disorders, Murdoch Childrens Research Institute, Melbourne, Australia; 9 Menzies School of Health Research, Charles Darwin University, Darwin, Australia; 10 National Organisation for Fetal Alcohol Spectrum Disorders, Adelaide, Australia; 11 Russell Family Fetal Alcohol Disorders Association, Cairns, Australia; 12 National Drug and Alcohol Research Centre, University of New South Wales, Sydney, Australia; 13 Nindilingarri Cultural Health Services, Fitzroy Crossing, Australia

**Keywords:** Fetal alcohol spectrum disorder, Diagnosis, Consensus

## Abstract

**Background:**

Fetal alcohol spectrum disorders (FASD) are underdiagnosed in Australia, and health professionals have endorsed the need for national guidelines for diagnosis. The aim of this study was to develop consensus recommendations for the diagnosis of FASD in Australia.

**Methods:**

A panel of 13 health professionals, researchers, and consumer and community representatives with relevant expertise attended a 2-day consensus development workshop to review evidence on the screening and diagnosis of FASD obtained from a systematic literature review, a national survey of health professionals and community group discussions. The nominal group technique and facilitated discussion were used to review the evidence on screening and diagnosis, and to develop consensus recommendations for the diagnosis of FASD in Australia.

**Results:**

The use of population-based screening for FASD was not recommended. However, there was consensus support for the development of standard criteria for referral for specialist diagnostic assessment. Participants developed consensus recommendations for diagnostic categories, criteria and assessment methods, based on the adaption of elements from both the University of Washington 4-Digit Diagnostic Code and the Canadian guidelines for FASD diagnosis. Panel members also recommended the development of resources to: facilitate consistency in referral and diagnostic practices, including comprehensive clinical guidelines and assessment instruments; and to support individuals undergoing assessment and their parents or carers.

**Conclusions:**

These consensus recommendations provide a foundation for the development of guidelines and other resources to promote consistency in the diagnosis of FASD in Australia. Guidelines for diagnosis will require review and evaluation in the Australian context prior to national implementation as well as periodic review to incorporate new knowledge.

## Background

Internationally, five different guidelines have been developed for the diagnosis of fetal alcohol syndrome (FAS) or fetal alcohol spectrum disorders (FASD), three of which were published by national health agencies or proposed for national implementation in North America [1-3]. Existing diagnostic guidelines for FASD have been developed using a range of approaches, including evidence-based consensus development methods [1,2] and studies of large clinical cohorts [4,5]. Although there is not international consensus on the diagnostic criteria for all FASD, more recent published guidelines [1,2,5] share some features based on concepts established in the original Institute of Medicine (IOM) diagnostic criteria [3] and the subsequent case-defined University of Washington (UW) 4-Digit Diagnostic Code [4].

Considerable gaps remain in the evidence base for diagnosis [6], which is likely to contribute to the variation in diagnostic practices [7] and the lack of international consensus on diagnosis. There is a need to improve service delivery and support health professionals’ capacity to diagnose FASD in Australia. Studies of FAS demonstrate inconsistency in diagnostic methods and a failure to diagnose the disorder [8,9], as found elsewhere [10,11]. Studies of Australian health professionals also indicate a need for training and resources to support practice [12,13], including locally-appropriate guidelines to improve diagnostic consistency and capacity [14,15].

The development of clinical guidelines is most appropriate where the potential impact of this is high [16]. In the context of considerable uncertainty about the diagnosis of FASD among Australian health professionals [13,14] and the absence of accurate estimates of FASD prevalence in Australia, the potential for national guidelines for FASD diagnosis to improve consistency in diagnostic practices [16,17], and identify gaps in management and prevention provide an important motivation for the development of national guidelines.

The use of systematic and transparent methods in the development of guidelines is important [18,19]. In addition, consistent with the need for a locally relevant approach to guideline development [16], Australian health professionals have raised concerns about adopting existing diagnostic guidelines for FASD, and highlighted the need for evidence of effectiveness in the local context [14]. Guideline development is often a qualitative process driven by the need to integrate diverse sources of evidence and multiple perspectives on factors that might influence guideline effectiveness, acceptability, suitability and utility in different clinical contexts [18-20].

The purpose of this study was to establish evidence-based consensus recommendations to support the development of guidelines for the diagnosis of FASD in Australia, including assessment methods and diagnostic criteria.

## Methods

Recommendations on the diagnosis of FASD were developed using systematic review and evaluation of the evidence based on the GRADE (Grading of Recommendations Assessment, Development and Evaluation) approach [21]. Due to the limited availability of local empirical evidence, this process predominantly involved evaluation and adaption of existing guidelines. This was based on the best available evidence and input from a panel of health professionals, consumers and others with relevant expertise. This consensus-based framework for developing recommendations is consistent with the recognised need to move beyond research evidence in the development of clinical guidelines [20].

The selected panel was small enough to enable exploration of reasons for disagreement or uncertainty, and large enough to produce reliable recommendations [20]. Study chief investigators (CB and EJE) purposively recruited 15 individuals with a range of relevant expertise (FASD diagnosis, research, education and advocacy) from 6 Australian states and territories. The 17 panel members included paediatricians, other health professionals, health researchers and consumer and community representatives. All panel members participated in the study design and were actively engaged in all components of the study.

**Figure 1:**
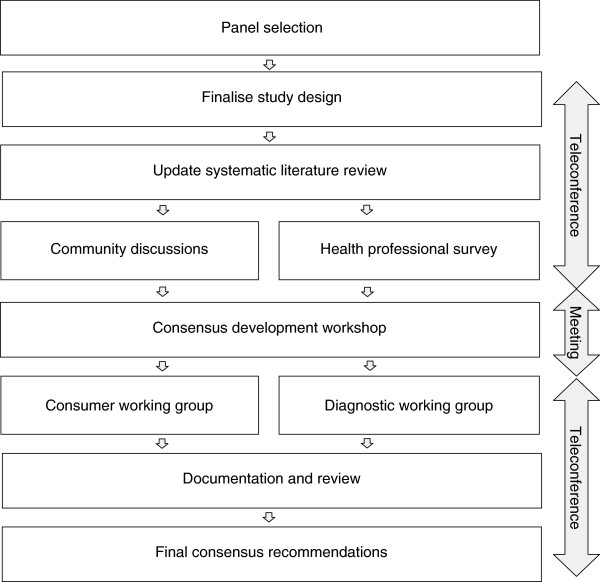
Study design and methods used to develop recommendations for the diagnosis of FASD in Australia.

Development of consensus recommendations for the diagnosis of FASD (Figure 1) was conducted over 22 months (August 2010 - May 2012) and included three main stages:

i. evidence collection and preliminary evaluation,

ii. a consensus development workshop and critical appraisal of evidence; and

iii. post-workshop documentation and review.

### Evidence collection and preliminary evaluation

We conducted: i) a systematic literature review on FASD screening and diagnosis which updated and expanded an existing review [6] to include literature published up to the 30^th^ September 2010; ii) a national consultation with health professionals using a modified Delphi process to identify their perceptions about adopting existing guidelines for diagnosis and agreement with existing screening and diagnostic criteria as described elsewhere [14,15,22]; and iii) discussions with women in the community about their perceptions of alcohol use in pregnancy and FASD. Findings were summarised and circulated to panel members for critical review before the workshop.

### Consensus development workshop

The nominal group technique, which is an established method for conducting structured group meetings [23,24], was used in combination with other informal methods to facilitate efficient problem exploration and consensus development. Structured exploration of key issues was particularly important given the diversity of the study panel. Workshop sessions involved evidence review, idea generation, large and small group discussion, and voting processes to develop consensus. Facilitated open group discussion sessions allowed participants to discuss and debate existing evidence; consider barriers to implementation and factors influencing local appropriateness; propose and clarify recommendations; and identify their logic and importance.

All panel members were invited to attend the 2-day workshop in July 2011 and 13 were able to attend. All 13 workshop participants had experience in FASD research and represented the range of expertise of the panel. Panel members who were unable to attend the workshop participated in the subsequent recommendation development and review processes.

### Post -workshop documentation and review

A diagnostic subgroup, including six medical practitioners (four paediatricians), met by teleconference to review the workshop outcomes and to complete and document recommendations. A three-member consumer subgroup also met by teleconference to review outcomes relating to consumer resources. All panel members then reviewed the consensus recommendations.

### Analysis

Consensus agreement was defined *a priori* as agreement by at least 70% of panel members. Recorded outcomes of formal and informal voting processes, flip chart records and field notes taken during open group discussions were used to analyse workshop findings. Qualitative descriptive analysis [25] of participant contributions in open discussions, based on identifying and categorising the underlying meaning of participant statements [26], was used to describe the main discussion content. To support the trustworthiness (credibility, dependability and confirmability) of the findings, all participants reviewed the workshop methods and findings to confirm that the recommendations were internally coherent and supported by the data [27].

The GRADE approach [21], which acknowledges the influence of a range of factors on the formulation of recommendations, was used to describe the strength [28,29] and quality of the evidence base [29] for each recommendation. A *strong recommendation* was made when the panel concluded there was clear evidence of balance between the desirable and undesirable effects of the strategy, or when there was little uncertainty about the benefits and harms of the strategy. A *conditional recommendation* was made where there was less certainty about the balance between desirable and undesirable effects. Strong recommendations were unlikely to be made in the absence of high quality evidence on costs and benefits. Evidence quality was rated high, moderate, low or very low based on its directness, likelihood of bias, consistency of findings, and likelihood that further research would modify confidence in the estimated effect [30]. This study was approved by the University of Western Australia Human Research Ethics Committee and the Western Australian Aboriginal Health Information and Ethics Committee. Written informed consent for participation was obtained from all panel members.

## Results

The panel noted the lack of specific, high quality, and locally relevant evidence on which to base recommendations about the diagnosis of FASD in Australia. Two consensus recommendations were developed on screening and referral, and five on diagnosis (Table 1).

**Table 1:** Summary of consensus recommendations for the diagnosis of FASD in Australia

**Area**	**Recommendation**
Population screening	We do not recommend population-based screening for FASD (GRADE: strong recommendation | low quality evidence)
Referral	We recommend the use of standard criteria for referral for specialist diagnostic assessment (GRADE: conditional recommendation | low quality evidence)
Diagnostic categories	We recommend the diagnostic categories of fetal alcohol syndrome, partial fetal alcohol syndrome and neurodevelopmental disorder-alcohol exposed for use in Australia (GRADE: conditional recommendation | low quality evidence)
Diagnostic criteria	We recommended that the diagnosis of fetal alcohol syndrome, partial fetal alcohol syndrome and neurodevelopmental disorder-alcohol exposed are based on the criteria summarised in Table 2 (GRADE: conditional recommendation | low quality evidence)
Diagnostic assessment methods	We recommend standard diagnostic assessment based on the comprehensive interdisciplinary UW approach to assessment (GRADE: conditional recommendation | low quality evidence)
Resources for implementation	We recommend the development of comprehensive resources to facilitate national implementation of standard diagnostic criteria and national case reporting (GRADE: conditional recommendation | low quality evidence)
Consumer information and support	We recommend that information and support are provided for individuals and their parents or carers during the diagnostic process (GRADE: conditional recommendation | low quality evidence)

FASD – fetal alcohol spectrum disorders;

GRADE – Grading of Recommendations Assessment, Development and Evaluation [21].

### Screening and referral

#### We do not recommend population-based screening for FASD (GRADE: strong recommendation | low quality evidence)

There are no reliable estimates of the population prevalence of FASD in most countries, including Australia. There is some evidence to suggest that the prevalence of FASD in high income countries may be as high as 2-5% [31]. However, effective screening for FASD requires a suitable screening test. Systematic reviews from Canada and New Zealand found limited information on the validity on different screening tests for FASD, insufficient evidence to justify population-based screening, and no single screening method for FASD suitable for all populations [6,32]. Similarly, our survey findings indicate little support for population-based screening, and highlight the absence of evidence on effectiveness [15]. Survey respondents and workshop participants also identified that the capacity for diagnosing and managing FASD in Australia is currently inadequate to support the introduction of population-based screening, and that in this context the harms of population-based screening outweigh its potential benefits.

There is some evidence that the benefits of screening may be greater among individuals in foster care, correctional environments and other high risk groups [33-37]. Evaluation of the feasibility, acceptability and effectiveness of screening for FASD in high risk groups is required before considering targeted screening in Australia.

#### We recommend the use of standard criteria for referral for specialist diagnostic assessment (GRADE: conditional recommendation | low quality evidence)

Both survey [15] and workshop participants endorsed the need for standard referral criteria to promote consistency and certainty in identifying the need for specialist assessment. Existing evidence-based diagnostic guidelines for FAS [1] and FASD [2] also recommend standard criteria for specialist referral. Studies of clinical cohorts [38] and high risk groups [33,39] provide evidence to support the use of standard criteria to identify the need for specialist diagnostic assessment, including prenatal alcohol exposure, growth deficit, central nervous system (CNS) dysfunction and developmental delay. Referral criteria for Australia should be adapted from existing consensus criteria [1,2], and evaluated in the local context.

Our conditional recommendation reflects the lack of direct high quality evidence of the effectiveness, costs and benefits of specific criteria for referral, and of whether implementation of standard referral criteria can improve awareness among health professionals of the need to assess prenatal alcohol exposure and consider FASD as a potential diagnosis. This recommendation places a high value on early diagnosis [40] and the demonstrated need for improved awareness among health professionals [8,12,13].

### Diagnosis

There is evidence that making a diagnosis of FASD, in combination with appropriate maternal services and support, can prevent the subsequent birth of affected children [35,41], reduce inappropriate management which may be harmful or counterproductive in individuals with FASD [42-44], and enable access to interventions that provide sustained benefit for affected individuals, their families and communities [40,45,46]. Workshop participants proposed that the diagnostic criteria used in either the UW [4] or Canadian [2] guidelines, or a combination of the two, should be used as a basis for the diagnosis of FASD in Australia. After reviewing the evidence, a formal vote established consensus support for combining elements of the UW and Canadian guidelines. Below are listed the five key recommendations for diagnosis in Australia.

#### We recommend the diagnostic categories of FAS, PFAS and neurodevelopmental disorder-alcohol exposed (ND-AE) for use in Australia (GRADE: conditional recommendation | low quality evidence)

The diagnostic categories recommended for use in Australia are FAS, PFAS and ND-AE. Despite the lack of established agreed diagnostic categories, FAS, PFAS, and alcohol-related neurodevelopmental disorder (ARND) are consistently identified as categories within the FASD spectrum [2-5,47-52]. The Australian category ND-AE reflects severe CNS dysfunction in the absence of facial anomalies and is broadly equivalent to the Canadian category ARND and the UW category static encephalopathy-alcohol exposed (SE-AE). Consistent with the Canadian guidelines, we do not recommend use of the UW diagnostic category of neurobehavioural disorder-alcohol exposed at this time, which requires evidence of moderate as opposed to severe CNS dysfunction. Although there is an extensive evidence base confirming prenatal alcohol exposure causes the full range of outcomes from moderate to severe CNS dysfunction [38,50-53] and a growing evidence base documenting significant CNS structural abnormalities among alcohol-exposed individuals with moderate dysfunction [38,47]; panel members identified the need for additional evidence to more fully evaluate the validity of diagnosis based on moderate CNS dysfunction, including significant dysfunction in only two domains or evidence of less severe dysfunction in three or more domains.

There was consensus that the diagnostic terminology for ARND should be modified to ensure that it describes the nature of the impairment, is meaningful to clinicians and consumers, and reflects the potentially unknown and multifactorial origins of neurodevelopmental disorders. The diagnostic term ND-AE uses the UW convention of designating a diagnostic category as alcohol exposed, rather than alcohol-related. Consistent with the UW and Canadian Guidelines, and evidence from the systematic review [6,54], the diagnostic category alcohol-related birth defects (ARBD) was not recommended for use.

#### We recommended that the diagnosis of FAS, PFAS and ND-AE are based on the criteria summarised in Table 2 (GRADE: conditional recommendation | low quality evidence)

**Table 2:** Recommended Australian FASD diagnostic categories and criteria

**Diagnostic criteria**^#^		** Diagnostic category**	
	**Fetal Alcohol Syndrome (FAS)**	**Partial Fetal Alcohol Syndrome (PFAS)**	**Neurodevelopmental Disorder-Alcohol Exposed (ND-AE)**
**Requirements for diagnosis**	Requires all 4 of the following criteria to be met:	Requires confirmed prenatal alcohol exposure, the presence of 2 of the 3 characteristic FAS facial anomalies at any age, and CNS criteria to be met:	Requires confirmed prenatal alcohol exposure and CNS criteria to be met:
**Prenatal alcohol exposure**	Confirmed or unknown	Confirmed	Confirmed
**Facial anomalies**	Simultaneous presentation of all 3 of the following facial anomalies at any age:	Simultaneous presentation of any 2 of the following facial anomalies^¤^ at any age:	No anomalies required^*^
	i. short palpebral fissure length (2 or more standard deviations below the mean)	i. short palpebral fissure length (2 or more standard deviations below the mean)	
	ii. smooth philtrum (Rank 4 or 5 on the UW Lip-Philtrum Guide^†^)	ii. smooth philtrum (Rank 4 or 5 on the UW Lip-Philtrum Guide^†^)	
	iii. thin upper lip (Rank 4 or 5 on the UW Lip-Philtrum Guide^†^)	iii. thin upper lip (Rank 4 or 5 on the UW Lip-Philtrum Guide^†^)	
**Growth deficit**	Prenatal or postnatal growth deficit indicated by birth length or weight ≤ 10th percentile adjusted for gestational age, or postnatal height or weight ≤ 10th percentile	No deficit required^*^	No deficit required^*^
**Central Nervous System (CNS) abnormality**	At least 1 of the following:		
	i. clinically significant structural abnormality (e.g. OFC ≤ 3rd percentile, abnormal brain structure), or neurological abnormality (seizure disorder or hard neurological signs); and/or		
	ii. severe dysfunction (impairment in 3 or more domains of function, 2 or more standard deviations below the mean) ^‡^		

OFC-occipital-frontal circumference. ^†^University of Washington Lip-Philtrum Guides: http://depts.washington.edu/fasdpn/htmls/lip-philtrum-guides.htm. ^*^Not required for diagnosis but may be present. ^#^Appropriate reference charts should be used, and other causes of growth deficit and CNS abnormality excluded. ^‡^Assessment of dysfunction based on evidence from standard validated assessment instruments interpreted by qualified professionals.

^¤^Based on the presence of 2 of the 3 characteristic FAS facial features, the observed impairments cannot be causally linked to prenatal alcohol exposure.

There is a growing evidence base for the UW diagnostic criteria [38,47,48,55-60], and there has been little validation of the Canadian criteria. However, participants recognised that there are a number of similarities between the UW and Canadian criteria for the diagnostic categories of FAS, PFAS and SE-AE/ARND (ND-AE). There was consensus agreement that the diagnostic criteria should include elements from both the UW and Canadian guidelines as outlined in Table 2, and that this would facilitate standardised reporting of diagnoses nationally.

Panel members identified a lack of evidence to compare the performance of criteria for CNS abnormality from the UW and Canadian guidelines, and that the specific criteria for establishing severe CNS damage or dysfunction was the greatest area of uncertainty in diagnosis, particularly in the absence of characteristic facial anomalies. Specifically, there was uncertainty about whether microcephaly alone was sufficient to indicate CNS damage, and whether moderate dysfunction was sufficient to indicate CNS damage. Consistent with the survey findings [22], use of the UW criteria for CNS abnormality, based on a significant structural abnormality or significant dysfunction in three or more domains, was recommended.

The requirement for confirmed prenatal alcohol exposure for the diagnosis of ARND or SE-AE in the Canadian and UW guidelines respectively was also recommended for the diagnosis of ND-AE. The panel acknowledged difficulties in the quantification of prenatal alcohol exposure associated with the availability of information on specific levels of exposure; variation in individual susceptibility; and implications for the interpretation of a safe level of exposure. Due to the range of factors that may modify the effect of prenatal alcohol exposure on growth [61-63], the diagnostic criteria for PFAS do not require the presence of a growth deficit, consistent with the UW and Canadian guidelines.

#### We recommend standard diagnostic assessment based on the comprehensive interdisciplinary UW approach to assessment (GRADE: conditional recommendation | low quality evidence)

To facilitate the use of valid and comprehensive assessment methods, workshop participants recommended the development of standard assessment protocols for all required examinations and investigations based on the UW interdisciplinary approach to diagnostic assessment, as also recommended in the Canadian guidelines. The UW diagnostic assessment approach was recommended based on its use of specific, quantifiable assessment methods, the accumulated evidence base resulting from its use [38,47,48,55,56], and endorsement of these methods by health professionals [14,22]. Panel members reached consensus agreement on essential components of the diagnostic assessment as listed in Table 3, all of which are assessed in the UW 4-Digit Diagnostic Code approach [4].

**Table 3:** Recommended Australian FASD diagnostic assessment content

**Recommended content**	**Content included on the UW FASD Diagnostic Form or New Patient Information Form**
History:	Yes
Family/social	Yes
Prenatal medical	Yes
Obstetric	Yes
Neonatal	Yes
Developmental	Yes
Academic	Yes
Current problems	Yes
Pre + post natal alcohol + other prenatal exposures	Yes
Paternal drinking	Yes
Drug and alcohol use in the child or individual	Yes
Early life trauma	Yes
Examination:	Yes
Growth	Yes
Head circumference	Yes
Dysmorphology	Yes
Central nervous system	Yes
Birth defects	Yes
Medical investigations	Yes
Diagnostic criteria	Yes
Exclusion of other diagnoses	Yes
Reporting final diagnosis by category	Yes
Results summary: strengths and areas of need	Yes
Follow-up and management plan	Yes

UW-University of Washington 4-Digit Diagnostic Code [4].

Given the lack of resources for specialised diagnostic services for FASD in Australia, a multidisciplinary approach to diagnosis with coordinated contributions from a range of professionals was considered more feasible for national implementation in the short term than the ideal interdisciplinary assessment model, where professionals from different disciplines work together in a structured and integrated team approach to diagnosis. The panel recommended that diagnostic assessment findings be directly applied to the identification of relevant diagnostic outcomes based on the Australian diagnostic criteria, and that the UW 4-digit code could also be derived if desired.

Consistent with nationally endorsed methods for the assessment of alcohol intake during pregnancy [64], panel members recommended standard assessment of prenatal alcohol exposure using the AUDIT-C [65]. This should be administered in combination with a clinical interview and case note review, where relevant, to obtain additional information about consumption patterns and timing. Both survey [14] and workshop participants noted a lack of evidence on which to evaluate the appropriateness of existing population references for the assessment of growth, facial anomalies and neurocognitive function, and the need for studies to determine culturally appropriate references for use in Australia.

#### We recommend the development of comprehensive resources to facilitate national implementation of standard diagnostic practices and national case reporting (GRADE: conditional recommendation | low quality evidence)

A comprehensive implementation plan was recommended to facilitate national adoption of standard diagnostic practices and development of systems for national surveillance of FASD. The implementation plan should include strategies and resources to: improve health professionals’ awareness of national diagnostic guidelines for FASD; facilitate adoption of standard diagnostic practices; provide training and support for health professionals, and establish national mechanisms for reporting and surveillance. Resources required would include comprehensive guidelines for diagnosis, standard instruments for referral and diagnosis, and training resources for health professionals.

#### We recommend that information and support are provided for individuals and their parents or carers during the diagnostic process (GRADE: conditional recommendation | low quality evidence)

Panel members recommended that culturally appropriate information and support services including counselling and advocacy should be available for individuals undergoing diagnostic assessment and their parents or carers. These should inform parents and carers or individuals about the diagnostic and management process and goals prior to the assessment, and provide on-going support. This recommendation recognises the importance of acknowledging the values and preferences of individuals undergoing assessment in clinical decision-making. Informed consent should be obtained and recorded prior to conducting the diagnostic assessment and communicating diagnostic findings to other individuals or external organisations. We recommend that parents and carers are involved in evaluating FASD resources and services as a part of standard quality improvement processes.

## Discussion

We propose evidence-based consensus recommendations for the diagnosis of FASD in Australia based on adaption of elements from the UW and Canadian guidelines. These recommendations were based on a review of evidence from published research and input from individuals with relevant expertise, including health professionals and consumer and community representatives. We recommend the three well established diagnostic categories of FAS, PFAS and ND-AE for use in Australia. The construct validity of the endorsed diagnostic categories is supported by the adoption of these categories in all published diagnostic guidelines for FASD internationally [2-5], despite minor differences in diagnostic criteria. However, there is not universal support for the validity of the diagnostic category of ND-AE/ARND or for the diagnosis of PFAS based on the presence of only two characteristic facial anomalies, and we acknowledge that the three diagnostic categories recommended for use do not represent the complete spectrum of disorders associated with prenatal alcohol exposure. The adequacy of evidence for diagnosis in these areas is still subject to debate.

Our systematic review of the literature demonstrated a lack of agreed diagnostic criteria for FASD and a lack of high quality evidence to enable direct comparison of different diagnostic criteria or evaluate their local applicability. These factors limited the use of the GRADE approach in developing recommendations, and our frequent use of conditional recommendations reflects uncertainty associated with current evidence base for diagnosis. Uncertainty was most notable for the Australian diagnostic criteria for ND-AE, where consensus was to use diagnostic criteria comparable with the UW guidelines for SE-AE and the Canadian guidelines for ARND in the requirement for evidence of severe CNS dysfunction, which is a more conservative approach than recommended in other guidelines [4,5]. Recommendations for Australia differ from the UW and Canadian guidelines in the lack of need to derive the 4-digit code; however, it can be derived if required.

Due to the lack of gold standard criteria for the diagnosis of FASD and categories within the spectrum, guideline development relied on a consensus-based approach. Primary limitations of consensus development panels include the potential for bias in the recruitment of panel members and in the participation of panel members in recommendation development. We attempted to minimise this bias by recruiting panel members from different states and territories and a range of professional backgrounds, use of an experienced facilitator, and use of formal consensus-development methods and structured group interaction to promote the involvement of all panel members.

The development of these recommendations was based on an integrated program of evidence collection and evaluation which aimed to facilitate extended engagement in a comprehensive critical evaluation process, used multiple sources of evidence, and consulted with health professionals and consumers to ensure recommendations were acceptable and locally appropriate. These processes allowed identification of uncertainty and reasons for disagreement, and provided a strong foundation for the content validity of these consensus-based recommendations.

National guidelines for diagnosis will require review and evaluation to establish their appropriateness and feasibility in the Australian context. This includes review by health professionals, policymakers, consumers and other stakeholders to identify issues that may affect performance, acceptability, cost-effectiveness and implementation [66]. The development of a comprehensive national implementation strategy, including specific resources to support implementation, is also required to facilitate adoption of national guidelines, improve diagnostic capacity and enhance the evidence base for diagnosis, surveillance, prevention, and management.

## Conclusion

National guidelines are required to promote consistent diagnostic practices for FASD in Australia and improve diagnostic capacity. These workshop recommendations provide a consensus-based foundation for the development of guidelines adapted from the UW and Canadian guidelines. Guidelines for diagnosis will require review and evaluation in the Australian context prior to national implementation as well as periodic review to incorporate new knowledge.

## Competing interests

The authors declare that they have no competing interests.

## Authors' contributions

CB, EJE and JMP designed the study and CB and EJE supervised the study. REW, AM, HJ and CB designed the workshop program, and all authors reviewed the study methods and procedures. HJ organised the workshop, and AM and REW facilitated the workshop. REW analysed the data and drafted the manuscript, and all authors critically reviewed the manuscript and approved the final version.

## Pre-publication history

The pre-publication history for this paper can be accessed here:

http://www.biomedcentral.com/1471-2431/13/156/prepub
